# No significant increase in thrombotic risk following the widespread adoption of TPO-RAs in ITP: A comparative study

**DOI:** 10.1016/j.gmg.2026.100107

**Published:** 2026-04-01

**Authors:** Yun Wang, Huiyuan Li, Yunfei Chen, Xiaofan Liu, Rongfeng Fu, Wei Liu, Feng Xue, Lei Zhang, Renchi Yang

**Affiliations:** a State Key Laboratory of Experimental Hematology, National Clinical Research Center for Blood Diseases, Haihe Laboratory of Cell Ecosystem, Tianjin Key Laboratory of Gene Therapy for Blood Diseases, CAMS Key Laboratory of Gene Therapy for Blood Diseases, Institute of Hematology & Blood Diseases Hospital, Chinese Academy of Medical Sciences & Peking Union Medical College, Tianjin 300020, China; b Tianjin Institutes of Health Science, Tianjin 301600, China

**Keywords:** Immune thrombocytopenia, Thrombopoietin receptor agonists, Thrombosis, Risk factors

## Abstract

In patients with primary immune thrombocytopenia (ITP), the risk of thrombosis associated with thrombopoietin receptor agonists (TPO-RAs) remains debated. This study compared the thrombosis occurrence in ITP patients before and after the launch of TPO-RAs in China, to explore the relationship between TPO-RAs and thrombosis in ITP patients. Data from 2005 to 2017 were obtained from an article published by the Blood Disease Hospital, Chinese Academy of Medical Sciences in 2018. The present study included 1915 hospitalized patients in the same center from January 2018 to February 2025. Among these, thrombosis events occurred in 33 cases (1.72%). The incidence of arterial thrombosis was higher than that of venous thrombosis. Male gender, age over 40 years old, hypertension, diabetes, smoking, and TPO-RAs were associated with thrombosis. Neither platelet count nor stage of ITP showed a significant effect on thrombosis risk. Although the utilization rate of TPO-RAs increased significantly after 2018, no corresponding significant change in thrombosis incidence was observed. These findings suggest that thrombosis in ITP patients results from the interplay of multiple risk factors. While the widespread use of TPO-RAs does not appear to increase the overall risk of thrombosis in ITP patients, caution is still advised when administering these agents to patients with pre-existing thrombosis risk factors.

## Introduction

Primary immune thrombocytopenia (ITP) is an autoimmune hematological disorder characterized by the reduction of platelet count. The pathogenesis of ITP is mainly due to the increase in platelet destruction mediated by platelet autoantibodies and T cells, as well as the impairment in platelet production [1]. Besides the elevated bleeding risk associated with thrombocytopenia, ITP patients also exhibit a higher incidence of thrombotic events compared with the general population [2]. Reported rates of arterial thrombosis range from 9.6 to 27.8 per 1000 person-years, while venous thrombosis occurs at an incidence of 4.1–6.7 per 1000 person-years [3], [4].

Thrombopoietin receptor agonists (TPO-RAs) are second-line treatment drugs for ITP. They promote megakaryocyte differentiation and maturation, thereby enhancing platelet production [5]. In recent years, TPO-RAs have been increasingly used in the management of ITP and are now established as key second-line treatments. However, whether TPO-RAs elevate the risk of thrombosis in ITP patients remains controversial, with considerable discrepancies observed across studies. A study by Lafaurie M, et al. demonstrated that TPO-RAs increases the risk of hospitalization due to thrombosis in patients with ITP [6]. Another study indicates that the occurrence of thrombosis caused by TPO-RAs is the result of multiple factors working together, rather than solely due to the effect of the drugs [7].

In December 2017, the first thrombopoietin receptor agonist (TPO-RA) was approved in China for the treatment of chronic immune thrombocytopenia. Due to its high efficacy and favorable administration profile, this class of agents has since been increasingly adopted in clinical practice. Taking 2018 as a reference point, this study compared the incidence of thrombosis and patterns of TPO-RA usage among ITP patients before and after this date. The aim was to elucidate whether the growing use of TPO-RAs is associated with an elevated thrombotic risk in ITP patients, providing a theoretical foundation to guide clinical medication decisions.

## Methods

This is a single center retrospective study. The eligible individuals were diagnosed as primary ITP according to the Chinese guidelines published in 2020 and American Society of Hematology 2019 guidelines for immune thrombocytopenia [8], [9]. Secondary ITP (hepatic disease-related thrombocytopenia, abnormal thyroid function, suspected connective tissue disease, malignant tumor, etc) was excluded. Patients with other malignant blood diseases or those whose diagnoses are unclear were also excluded. We collected the basic information of inpatients with primary ITP from January 2018 to February 2025, including gender, age, smoking, underlying diseases, history of thrombosis, disease course, previous treatments, platelet count, etc. The number of patients who receive TPO-RAs was defined as patients who were taking TPO-RAs before admission or initiated TPO-RAs after admission. Patients in the thrombotic group were defined as those who developed thrombosis after exposure to TPO-RAs; those with a prior history of thrombosis were not included in this category. The diagnosis of thrombosis required clear confirmation through ultrasound and computed tomography angiography (CTA). The diagnosis of cerebral infarction requires a magnetic resonance examination. Data prior to 2018 are from a previously published article of this center, which collected the information of ITP patients from December 2005 to December 2017 [10]. This study also enrolled ITP patients who were hospitalized at the same center. Information on age, sex, underlying diseases, personal history, and treatment history was collected. For patients who developed thrombosis, additional data on thrombotic events were collected, including the site of thrombosis, platelet count at the time of thrombosis, clinical symptoms, and imaging findings. The two studies shared the same diagnostic criteria for ITP and collected comparable clinical information. Comparing the differences in thrombosis of ITP patients before and after 2018. The purpose is to compare whether the incidence of thrombosis in ITP patients has changed after 2018, and whether this change is related to the wide-scale use of TPO-RAs in China.

Statistical analysis was performed with IBM SPSS Statistics 26. Continuous variables are described with the median (M) and interquartile range (IQR). The comparison between the rates was conducted using the Chi-square test. Univariate analysis was used to compare the thrombosis group and the non-thrombosis group in order to identify the risk factors for thrombosis. *P* < 0.05 is considered statistically significant.

## Results

A total of 2114 hospitalized patients diagnosed with immune thrombocytopenia were identified between January 2018 and February 2025. After excluding individuals with unclear diagnoses or secondary thrombocytopenia, 1915 patients with primary ITP were enrolled who were hospitalized at the Blood Disease Hospital, Chinese Academy of Medical Sciences. Among them, 719 (37.55%) were male and 1196 (62.45%) were female, with a median age at diagnosis of 41 years. The cohort comprised 31.49% newly diagnosed ITP cases, 27.20% persistent ITP cases, and 41.31% chronic ITP cases. Thrombotic events occurred in 33 (1.72%) patients following the detection of thrombocytopenia. Of these, 18 (54.54%) were male, and one case (3.03%) occurred in a patient under 18 years of age. The incidence of arterial thrombosis was 1.15% (22/1915), venous thrombosis was 0.37% (7/1915), and combined arterial and venous thrombosis was 0.21% (4/1915). Platelet counts at the time of thrombotic events exhibited considerable variation, ranging from 2 × 10⁹/L to over 800 × 10⁹/L. More clinical characteristics are summarized in Table 1. Reviewing the research conducted by our center on the thrombosis conditions of ITP patients from 2005 to 2017, the thrombosis formation rate was 1.43% (46/3225). The data for the period from 2005 to 2017 is derived from the previous study [11].

**Table 1:** Clinical Characteristics of the 33 ITP Patients with Thrombotic Events.

	**Thrombosis group(n = 33)**
Male/Female	18/15
Age (M/IQR)	55/19.50
Stages (%)	
Newly diagnosed ITP	5 (15.15%)
Persistent ITP	14 (42.42%)
Chronic ITP	14 (42.42%)
Hypertension (%)	16(48.48%)
Diabetes mellitus (%)	9 (27.27%)
Smoking (%)	9 (27.27%)
Treatments (%)	
Glucocorticoids	16 (48.48%)
TPO-RAs	21 (63.63%)
Splenectomy or embolization	0 (0%)
Plateletcounterwhenthrombosis occurs (× 109/L) (%)	
0–30	14 (42.42%)
31–50	3 (9.09%)
51–100	3 (9.09%)
> 100	8 (24.24%)
Arterial thrombosis (%)	22 (66.67%)
Venous thrombosis (%)	7 (21.21%)
Arteriovenous thrombosis (%)	4 (12.12%)
Events with hemorrhage(%)	2 (6.06%)

**Fig. 1:**
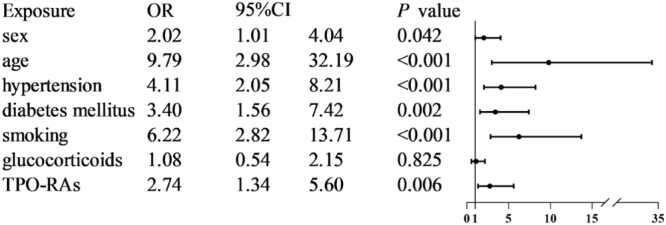
Univariate binary logistic regression analysis between the thrombus group and the non-thrombus group.

Our results demonstrated a higher incidence of arterial thrombosis compared to venous thrombosis in a Chinese population. The most common sites of thrombosis were the cerebral arteries, followed by the lower extremity veins and coronary arteries. Additional information can be found in Table 2. Thrombotic events occurred across all phases of ITP, with no statistically significant differences in incidence among disease stages (Table 3). Comparative analysis between the thrombosis and non-thrombosis groups revealed several significant differences. A higher proportion of male patients was observed in the thrombosis group (54.55% *vs* 37.25%, OR=2.02, 95%CI 1.01–4.04, *P* = 0.042). Patients over 40 years of age were also more prevalent in the thrombosis group (90.91% *vs* 50.53%, OR=9.79, 95%CI 2.98–32.19, *P* < 0.001). Comorbidities such as hypertension (48.48% *vs* 18.65%, OR=4.11, 95%CI 2.05–8.21, *P* < 0.001) and diabetes mellitus (27.27% *vs* 9.94%, OR=3.40, 95%CI 1.56–7.42, *P* = 0.002) were more frequent in the thrombosis group, as was a history of smoking (27.27% *vs* 5.69%, OR=6.22, 95%CI 2.82–13.71, *P* < 0.001). Both groups had similar glucocorticoid usage rates, with no statistically significant difference (thrombosis group *vs* non-thrombosis group, 48.48% *vs* 46.55%, OR=1.08, 95%CI 0.54–2.15, *P* = 0.825). None of the patients in the thrombosis group underwent splenectomy or splenic embolization, compared to 39 (2.07%) in the non-thrombosis group. There was no statistically significant difference between the two groups. (Fig. 1; Table 4)

**Table 2:** Thrombus classification.

**Thrombotic site**	**Number**
Brain arteries	16
Coronary artery	5
Pulmonary artery	3
Lower extremity arteries	3
Lower extremity veins	9
Portal vein, superior mesenteric vein	1
Right common carotid artery, right internal carotid artery, right external carotid artery, right subelavian artery, right vertebral artery	1

**Table 3:** The ITP stages of the thrombosis group and the non-thrombosis group.

	**Newly diagnosed ITP**	**Persistent ITP**	**Chronic ITP**	**χ² value**	***P*****-value**
Thrombosis group (n = 33)	5(15.15%)	14(42.42%)	14(42.42%)	3.76	0.152
Non-thrombosis group (n = 1882)	598(31.77%)	507(26.94%)	777(41.29%)

**Table 4:** Evaluation of thrombosis risk factors in patients with ITP.

	**Thrombosis group group (n = 33)**	**Non-thrombosis (n = 1882)**	**χ²value**	**P-value**
Sex(%)			4.14	0.042
Male	18(54.55%)	701(37.25%)		
Female	15(45.45%)	1181(62.75%)		
Age(%)			21.16	< 0.001
≤ 40	3(9.09%)	931(49.47%)		
> 40	30(90.91%)	951(50.53%)		
Hypertension(%)	16(48.48%)	351(18.65%)	18.63	< 0.001
Diabetes mellitus(%)	9(27.27%)	187(9.94%)	8.81	0.003
Smoking(%)	9(27.27%)	107(5.69%)	22.91	< 0.001
Treatments(%)				
Glucocorticoids	16(48.48%)	876(46.55%)	0.05	0.825
Splenectomy or embolization	0(0%)	39(2.07%)		1.000
TPO-RAs	21(63.63%)	734(39.00%)	8.24	0.004

Patients in the thrombosis group exhibited a significantly higher usage of TPO-RAs compared to those in the non-thrombosis group (63.63% *vs* 39.00%, OR=2.74, 95%CI 1.34–5.60, *P* = 0.006, Fig. 1). Further analysis of the demographic characteristics of the TPO-RAs group and the non-TPO-RAs group revealed that there were no significant differences between the two groups in terms of age and gender composition, incidence of and diabetes mellitus, and smoking history (Table 5).

**Table 5:** Comparison of characteristics between the population using TPO-RAs and the population not using TPO-RAs.

	**TPO-Ras (n = 755)**	**non-TPO-Ras (n = 1160)**	**χ²value**	**P-value**
Sex(%)			0.12	0.733
Male	287(38.01%)	432(37.24%)		
Female	468(61.99%)	728(62.76%)		
Age(%)			0.81	0.368
≤ 40	359(47.55%)	576(49.66%)		
> 40	396(52.45%)	584(50.34%)		
Hypertension(%)	140(18.54%)	227(19.57%)	0.31	0.577
Diabetes mellitus(%)	90(11.92%)	106(9.14%)	3.85	0.050
Smoking(%)	45(5.96%)	71(6.12%)	0.02	0.886

TPO-RAs began to be launched in China in December 2017. Prior to 2018, recombinant human thrombopoietin (rhTPO) was the main thrombopoiesis-stimulating agent available. We compared the utilization of TPO-RAs before and after 2018. Among ITP patients hospitalized in our institution between 2005 and 2017, the usage rate of recombinant human thrombopoietin and TPO-RAs was 23.50%. From 2018–2025, the usage rate of TPO-RAs alone reached 39.43%. This increase was statistically significant (*P* < 0.001) (Table 6). Notably, although TPO-RA usage rose significantly after 2018, the overall incidence of thrombotic events did not increase (Table 7). Furthermore, no significant difference was observed in the distribution of thrombosis types between the two periods (Table 8).

**Table 6:** Comparison of TPO-RAs/rhTPO usage before and after 2018.

	**2005–2017**^a^	**2018–2025**	**Total**	**χ²value**	**P-value**
TPO-RAs	521(23.50%)	755(39.43%)	1276	122.09	< 0.001
Not using TPO-RAs	1696(76.50%)	1160(60.57%)	2856		
Total	2217	1915	4132		

a The data from 2005 to 2017 represent the total number of people who used TPO-RAs and/or rhTPO.

**Table 7:** Comparison of thrombosis incidence rates before and after 2018.

	**2005–2017**	**2018–2025**	**Total**	**χ²value**	**P-value**
Thrombosis	46(2.07%)	33(1.72%)	79	0.68	0.421
No thrombosis	2171(97.93%)	1882(98.28%)	4053		
Total	2217	1915	4132

**Table 8:** Comparison of thrombus types before and after 2018.

	**2005–2017**	**2018–2025**	**Total**	**χ²value**	**P-value**
Arterial thrombosis(%)	36(78.26%)	22(66.67%)	58	1.52	0.509
Venous thrombosis(%)	7(15.22%)	7(21.21%)	14		
Arteriovenous thrombosis(%)	3(6.52%)	4(12.12%)	7		
Total	46	33	79		

## Discussion

This single-center study included a total of 1915 inpatients with primary ITP. Among them, 33 patients developed thrombosis after the diagnosis of thrombocytopenia, yielding an overall thrombosis incidence of 1.72%. Arterial thrombosis was more frequent than venous thrombosis. The occurrence of thrombosis had no significant relationship with the clinical phase of ITP. When thrombosis occurred, the platelet count varied greatly, ranging from < 10 × 10^9/L to > 800 × 10^9/L. Risk factors significantly associated with thrombosis included male sex, age over 40 years, hypertension, diabetes mellitus, smoking history, and prior use of TPO-RAs. No significant differences were observed between the thrombosis and non-thrombosis groups regarding the use of glucocorticoids or rates of splenectomy/embolization. Although the utilization rate of TPO-RAs increased significantly after 2018, no corresponding increase in the incidence of thrombosis was observed. Furthermore, no significant differences were detected in baseline characteristics or thrombotic risk between patients treated with TPO-RAs and those who were not.

Over the past few decades, extensive research has focused on the occurrence of thrombosis in patients with ITP. These studies have consistently shown that ITP is not merely a bleeding disorder, but also carries an increased risk of thrombosis. A British cohort study, which included 1070 ITP patients and 4280 non-ITP controls matched for age, gender, and primary care practice, demonstrated that the proportions of ITP patients who developed venous, arterial, and arteriovenous thrombosis for the first time were 2.9%, 4.1%, and 6.1%, respectively. The adjusted risk ratios for thrombosis in ITP patients compared to non-ITP controls were 1.58, 1.37, and 1.41, respectively [12]. Saac Goncalves et al. conducted a retrospective cohort study from January 1st 2011 to October 30th 2022, following up on 220 adult patients with ITP. The incidence of arterial thrombosis was 0.66 per 100 person-years, and the incidence of venous thrombosis was 2.05 per 100 person-years [13]. A comparison of thrombosis incidence in the same center from two distinct periods (2005–2017 and 2018–2025) revealed no significant difference, with arterial thrombosis remaining the predominant form in both periods. This finding aligns with the aforementioned UK study [12]. Notably, our analysis suggests that treatment modalities for ITP over the past two decades have not affected the incidence of thrombosis. Additionally, a Chinese study further supports the notion that ITP patients are at a heightened risk of thrombosis [14]. Notably, arterial thrombosis is more prevalent than venous thrombosis in this population, a trend that is consistent with findings in the UK cohort.

The causes of thrombosis in ITP can mainly be classified into three aspects: the disease itself of ITP, the patient's own condition, and the related treatments for ITP [15], [16]. These three factors interact with each other and promote each other. In ITP patients, elevated levels of platelet and red blood cell-derived microparticles, along with acquired resistance to protein C, contribute to a hypercoagulable state [17]. Additionally, the endothelium is often activated or injured, and platelets exhibit a heightened activation profile [18]. Many factors together contribute to the pro-coagulation state of ITP. Factors such as age, secondary ITP, multi-line therapy, TPO-RAs, and splenectomy are associated with thrombosis in ITP [13], [19]. A study followed up 303 newly diagnosed ITP patients showed that smoking, hypertension, male, history of thrombosis, and atrial fibrillation (AF) were significantly associated with the occurrence of thrombosis. Lupus anticoagulant positivity or ITP treatment did not increase the risk of thrombosis [20]. In our study, no patient in the thrombosis group had undergone splenectomy, whereas only 2.07% of patients in the non-thrombosis group had a history of splenectomy or splenic embolization. With the advent of newer pharmacological agents, the role of splenectomy, an invasive procedure, has diminished in the management of chronic ITP. Based on previous clinical studies [21], [22], [23], clinicians increasingly avoid or postpone splenectomy for patients with high thrombosis risk factors and other postoperative complications [24], [25]. Therefore, the occurrence of thrombosis events due to splenectomy is reduced in patients with ITP. In the research conducted at this center, whether before 2018 or after 2018, glucocorticoids were not identified as a risk factor for thrombosis, which might be related to their short-term application. In this study, most thrombotic events were detected after the onset of symptoms and subsequent targeted examinations. However, with the current routine venous thromboembolism (VTE) assessment for hospitalized patients and increased vigilance among clinicians regarding the risk of thrombosis in this population, the number of patients with asymptomatic thrombosis identified in recent years has increased.

TPO-RA, as a second-line treatment for ITP, has been widely adopted due to its remarkable efficacy and convenient administration, establishing them as a cornerstone of second-line therapy [26]. The sustained efficacy rate of TPO-RAs is approximately 40–60% [27], [28], [29]. Among adult patients with persistent or chronic ITP who achieved a stable complete response to TPO-RAs, more than 50% were able to discontinue any ITP treatment without any significant severe bleeding after one year [30]. One main concern regarding TPO-RA therapy is the potential increased risk of thrombosis. Long-term data from a study on eltrombopag reported thrombotic events in 6% of patients [31]. Another study on romiplostim found a drug-related thrombosis incidence of 2.9%, which did not increase with extended treatment duration and showed no significant difference compared to the placebo group [32]. However, two meta-analyses have shown that TPO-RAs do not increase the risk of thrombosis [33], [34].

A network meta-analysis compared the efficacy and safety of avatrombopag, romiplostim, hetrombopag, and eltrombopag. SUCRA analysis showed that among the four drugs, avatrombopag had the best safety profile, followed by eltrombopag, romiplostim, and hetrombopag. Based on adverse event reports, avatrombopag has exhibited a low incidence of hemorrhagic and thrombotic events since its launch, along with a relatively limited number of concurrent adverse events [35]. Another study showed that the SUCRA value for thrombotic events associated with romiplostim was 33.2, which was significantly lower than that of other small-molecule TPO-RAs, while the SUCRA value for eltrombopag was 62.1. These data indicate that romiplostim is associated with a lower risk of thrombosis than eltrombopag [36]. Our research results show that TPO-RAs are associated with thrombosis in ITP patients. We compared the use of TPO-RAs before and after the end of 2017. Of note, the data collected from 2005 to 2017 in the previous study represented the combined utilization rate of rhTPO and TPO-RAs. On one hand, TPO-RAs were not yet available in China before 2017, and only a limited number of patients received the treatment, resulting in extreme imbalance in sample sizes between the two time points and consequently reduced statistical power. Moreover, the inclusion of patients who received rhTPO in the previous data does not affect the conclusions of this study. On the other hand, both rhTPO and TPO-RAs are thrombopoietin receptor agonists, which can, to some extent, reflect the risk of thrombosis associated with an increase in platelet count. Although TPO-RA utilization has risen markedly since 2018, the overall incidence of thrombosis in our cohort did not increase correspondingly. This indicates that thrombosis in ITP is the result of multiple factors working together. The widespread use of TPO-RAs does not significantly increase the risk of thrombosis.

This study longitudinally compared the incidence of thrombosis among ITP patients at a single center before and after 2018—the year marking the widespread introduction of TPO-RAs in China. No significant change in thrombotic events was observed following the adoption of TPO-RAs. To our knowledge, this is the first study to evaluate the impact of TPO-RAs on thrombotic risk in ITP patients from a temporal perspective. Several limitations of this study should be acknowledged. First, as only inpatients were included, outpatient cases were not captured, which might have led to the omission of some ITP patients who did not meet the hospitalization criteria but developed thrombosis. Second, no post-discharge follow-up was conducted for patients treated with TPO-RAs, which may have resulted in an underestimation of thrombosis incidence associated with these agents; future prospective cohort studies are warranted to address this gap. Third, the single-center design may introduce selection bias. Multicenter studies are needed to enhance the generalizability of the findings. Finally, due to frequent switching between TPO-RAs after initial treatment failure, it was not feasible to attribute thrombotic risk to any specific agent. Therefore, subgroup analysis among different TPO-RAs was not performed.

## Conclusion

By analyzing the incidence of thrombosis in ITP patients over the past two decades and comparing the rates before and after the introduction of TPO-RAs in China in 2018, this study demonstrates that the widespread use of TPO-RAs does not lead to an overall increase in thrombotic events among ITP patients. However, given that TPO-RA treatment is associated with thrombosis, caution is advised when administering these agents to patients with pre-existing thrombotic risk factors.

## Ethics approval

The study was approved by the Ethics Review Board of the Institution of Blood Diseases Hospital, Chinese Academy of Medical Sciences (reference numbers: NKRDP2023006-EC-2). The human information and medical records used in this study were obtained from previous clinical diagnoses and treatments. Waiving informed consent will not adversely affect their individual rights or health. This study adheres to the ethical principles of the Declaration of Helsinki. Therefore, the ethics committee approved an exemption from the requirement to obtain signed informed consent.

## Declaration of Competing Interest

The authors declare that they have no known competing financial interests or personal relationships that could have appeared to influence the work reported in this paper.

## Data Availability

No datasets were generated or analysed during the current study.
